# Double-blind, placebo-controlled, randomized phase II study of TJ-14 (Hangeshashinto) for infusional fluorinated-pyrimidine-based colorectal cancer chemotherapy-induced oral mucositis

**DOI:** 10.1007/s00280-015-2767-y

**Published:** 2015-05-17

**Authors:** Chu Matsuda, Yoshinori Munemoto, Hideyuki Mishima, Naoki Nagata, Mitsuru Oshiro, Masato Kataoka, Junichi Sakamoto, Toru Aoyama, Satoshi Morita, Toru Kono

**Affiliations:** Department of Surgery, Osaka General Medical Center, Osaka, Japan; Department of Surgery, Fukui Saiseikai Hospital, Fukui, Japan; Cancer Center, Aichi Medical University, Nagakute, Japan; Kitakyushu General Hospital, Kitakyushu, Japan; Toho University, Sakura Hospital, Sakura, Japan; Department of Surgery, Nagoya Medical Center, Nagoya, Japan; Tokai Central Hospital, Kakamigahara, Japan; Department of Gastrointestinal Surgery, Kanagawa Cancer Center, 2-3-2 Nakao, Asahi-ku, Yokohama, 241-8515 Japan; Department of Biostatistics and Epidemiology, Yokohama City University, Yokohama, Japan; Advanced Surgery Center, Sapporo Higashi-Tokushukai Hospital, Sapporo, Japan

**Keywords:** Hangeshashinto, TJ-14, Colorectal Cancer, Oral mucositis

## Abstract

**Purpose:**

Hangeshashinto (TJ-14, a Kampo medicine), which reduces the level of prostaglandin E2 and affects the cyclooxygenase activity, alleviates chemotherapy-induced oral mucositis (COM). We conducted a double-blind, placebo-controlled, randomized comparative trial to investigate whether TJ-14 prevents and controls COM in patients with colorectal cancer.

**Methods:**

Ninety-three patients with colorectal cancer who developed moderate-to-severe COM (WHO grade ≧1) during any cycle of chemotherapy using FOLFOX, FOLFIRI, and/or XELOX treatment were randomly assigned to receive either TJ-14 (*n* = 46) or placebo (*n* = 47). Patients received the administration of placebo or TJ-14 for 2 weeks at the start of the next course of chemotherapy. Patients were assessed three times per week for safety and for COM incidence and its severity using the WHO grading.

**Results:**

Ninety eligible patients (TJ-14; 43, placebo; 47) per protocol set analysis were included in the analysis after the key-opening. Although the incidence of grade ≧2 oral mucositis was lower for patients treated with TJ-14 compared to those treated with placebo, there was no significant difference (48.8 vs. 57.4 %; *p* = 0.41). The median duration of grade ≧2 mucositis was 5.5 versus 10.5 days (*p* = 0.018). No difference in other treatment toxicity was observed between the two groups, and patients exhibited high compliance in dosing administration.

**Conclusion:**

The present study results did not meet the primary endpoint. However, TJ-14 demonstrated a significant effect in the treatment of grade ≧2 mucositis in patients with colorectal cancer compared to the placebo.

## Introduction

Oral mucositis is a common complication of systemic high-dose chemotherapy as well as radiotherapy for cancer [1, 2]. Oral mucositis is associated with higher risk of infection, pain, chemotherapy dose reduction, and infection-related death. Severe mucositis impairs oral and esophageal functions and seriously affects nutrition and quality of life of the patients. Consequently, oral mucositis can result in therapeutic noncompliance or become a dose-limiting toxicity that requires treatment modifications or interruption and eventually affect the outcome of cancer therapy. Several reviews and clinical practice guidelines have confirmed the need for increased emphasis on the management of mucositis [3–5].

Despite the attempts to reduce cancer chemotherapy-related mucositis, there has been no standard efficacious prophylactic therapy. Treatment is mostly supportive, consisting of good oral hygiene, mouthwashes, and analgesia [2].

Evidence from randomized trials suggests that the use of oral ice tips before chemotherapy improves oral mucositis by decreasing blood flow and decreases drug absorption [6–8]. Other clinical trials attempting to evaluate various strategies to prevent or reduce chemotherapy-induced mucositis, enrolled only small numbers of patients in their studies, and were thus inconclusive [9–13]. Only in two studies of large randomized double-blind, placebo-controlled trials were treatments reported to be effective against mucositis. One trial involved 326 women with breast cancer receiving an anthracycline regimen, and a new agent AES-14 was reported to significantly reduce the incidence of mucositis, although this result has not yet been reproduced or reconfirmed [14]. The other study investigating the effect of human epidermal growth factor for mucositis in head and neck cancer patients, proved significant reduction in severe oral mucositis receiving radiotherapy and chemotherapy [15]. Despite those results, it is still unclear whether there is any definite evidence for the prevention and treatment of cancer chemotherapy-induced oral mucositis to date.

The mechanism of chemotherapy-induced mucositis has been investigated and a report speculated that the cyclooxygenase pathway mediates tissue injury and pain through upregulation of pain-evoking prostaglandin E2 and proinflammatory cytokines [16, 17]. TJ-14 (Hangeshashinto), a traditional Japanese herbal (Kampo) medicine, was shown to significantly decrease production of prostaglandin E2 in human oral keratinocytes in hamsters. The gene expressions of cyclooxygenase 2, cytosolic phospholipase A2, and prostaglandin E synthase were also down-regulated by exposure to TJ-14 [18]. A clinical study demonstrated that topical administration of TJ-14 significantly reduced grade 3/4 mucositis in 13 out of 14 patients with oral mucositis, and TJ-14 was considered to have therapeutic effect on chemotherapy-induced oral mucositis via down regulation of proinflammatory prostaglandins in the cyclooxygenase pathway [19].

Given those biochemical and in vivo study findings, in the present study, the efficacy of TJ-14 for the prevention and/or treatment of chemotherapy-induced oral mucositis was investigated in a randomized double-blind, placebo-controlled exploratory clinical trial in colorectal cancer patients.

## Patients and methods

### Study design

A multi-institutional, double-blind, placebo-controlled randomized phase 2 trial was performed in patients receiving chemotherapy for advanced colorectal cancers in Japan. Patients who developed WHO grade ≧1 oral mucositis during the first screening cycle of chemotherapy were eligible for randomization to the study. Eligible patients were centrally randomized to receive either TJ-14 or placebo during their next second cycle of chemotherapy. Patients were stratified by chemotherapy regimen before randomization in a 1:1 ratio. A matched placebo, specially made and prepared, was utilized to maintain blinding. The primary objective of this study was to determine the efficacy and safety of TJ-14 compared to placebo when used to reduce the incidence of severe (WHO grade ≧3) oral mucositis associated with mucotoxic FOLFOX, FOLFIRI, or XELOX chemotherapy for advanced colorectal cancer. Secondary objectives were to determine the treatment effect of TJ-14 compared to placebo on the duration of WHO grade ≧2 oral mucositis, and the worst oral mucositis grade throughout the protocol therapy. Time to disappearance of oral mucositis was also compared between the two treatments.

### Ethical considerations

The study data and informed consent were obtained in accordance with the Declaration of Helsinki and were approved by the Ethics Review Board of each participating institution.

### Study drug

Both TJ-14 and placebo were administered at a dose of 2.5 g X3 times per day for a total daily dose of 7.5 g. The placebo formulation matched the texture, flavor, and other characteristics of the active drug. Patients were advised to dissolve 2.5 g of TJ-14 or placebo in 50 ml of drinking water, divided it into twice or three times in an oral cavity. They rinsed their oral cavity with it three times daily. In principal, the patients were trained and validated by the physician at out patient’s clinic. Study drug treatment began on the first day of chemotherapy and continued for 14 days. Patients followed oral care instruction throughout the treatment before the next course of chemotherapy began. No other prophylactic mouthwashes or treatment for mucositis were allowed in this clinical trial.

### Study assessments

Signs and symptoms of oral mucositis were assessed by the investigator 3 times per week on nonconsecutive days during the screening cycle and during treatment cycle 1 and 2 (on days 3, 5, 7, 9, 11, and 14) and additionally on days 16 and 18 of each cycle. The WHO oral mucositis scale was used to assess the severity of oral mucositis. Oral assessment continued for the first 3 weeks or until mucositis returned to grade 0. Additionally, patients themselves reported their ability to eat foods.

Safety was assessed throughout the study by physical examination, including inspection of oral tissues, hematology and serum chemistry laboratory tests, and adverse event reporting. Any adverse event, whether or not related to the study drug, was reported with date and time of onset, severity, pattern, action taken, and outcome. If the adverse event had not resolved at the time the case report forms were collected, a follow-up report was provided at a later date. If no follow-up report was provided, the investigator had to give a justification. All adverse events were followed until they either resolved or the investigator determined that the event was no longer clinically significant. Tumor response to chemotherapy was also evaluated every month. RECIST ver. 1.1 criteria were used to assess the efficacy of chemotherapy to the target lesion.

### Statistical analyses

Eligible patients were randomly assigned on a 1:1 ratio to receive either TJ-14 or placebo. After checking patient eligibility, randomization was carried out centrally at the data center using minimization with the stratification factors, including chemotherapy regimen (adjuvant chemotherapy for resected tumor/chemotherapy for advanced and/or metastatic cancer), previous history for any type of stomatitis treatment (yes/no), age (<60/≧60), and institution.

The primary endpoint of this study was the incidence of persisting grade ≧2 oral mucositis for over 1 week, which was compared between the TJ-14 and placebo groups using the Chi-squared test.

Assuming that the incidence of oral mucositis of grade 2 or greater was 10 % in the TJ-14 group and 35 % in the placebo group, 42 patients per group were required to compare the two treatment groups using Chi-squared test with a two-sided *α* = 0.10 and a power of 80 %. Thus, to account for possible dropouts, a target sample size of 90 patients was required. The secondary endpoints included the duration of grade ≧2 oral mucositis, and the worst oral mucositis grade throughout the protocol therapy, and time to disappearance of oral mucositis. The time-to-event data were analyzed using the Kaplan–Meier method and compared between the treatments groups using the log-rank test. All p values were two-sided. Statistical analyses were performed with SAS for Windows, release 9.3 (SAS Institute, Cary, NC).

## Results

### Patients

Of 707 patients in 19 participating institutions who were receiving 5-FU-based chemotherapies for colorectal cancers, 93 who developed WHO grade ≧1 oral mucositis during the screening cycle and provided informed consent were randomized to either TJ-14 (*n* = 46) or placebo (*n* = 47) after the first treatment cycle. Among those, projected administration could not be started in three patients in the TJ-14 group and thus they were excluded from the study. Baseline demographics and disease characteristics for the per protocol set (PPS) population are shown (Table 1). 55.5 % were men and 44.5 % were women; median age was 67 years (range 29–85 years). All patients had histologically confirmed adenocarcinoma of the colon (69.0 %) and rectum (31.0 %). Slight disparity in gender (*p* = 0.186) and performance status (*p* = 0.178) distribution was noticed between the two PPS randomized groups. The majority of patients received FOLFOX (40 %), FOLFIRI (30 %), or XELOX (7.8 %), and treatment groups were balanced for chemotherapy regimen (Table 2). No patient received radiation therapy before enrollment. No patient was enrolled in the study if there was any clinical evidence of another active oral mucosal disease at baseline. More than 90 % of the PPS patients completed the study, with little difference in the rate of discontinuation or postponement during treatment with TJ-14 or placebo.

**Table 1:** Patient characteristics of the TJ-14 and placebo groups

Treatment	TJ-14 (*n* = 43)	Placebo (*n* = 47)	*p* value
Sex			
Male	27 (62.8 %)	23 (48.9 %)	0.186
Female	16 (37.2 %)	24 (51.1 %)	
Age			
Median	67.0	67.0	0.376
Range	49.0–84.0	29.0–85.0	
PS			
0	34 (79.1 %)	42 (89.4 %)	0.178
1	9 (20.9 %)	5 (10.6 %)	
Location			
Colon	29 (67.4 %)	33 (70.2 %)	0.777
Rectum	14 (32.6 %)	14 (29.8 %)	
Status			
Adjuvant	10 (23.3 %)	14 (29.8 %)	0.484
Advanced	33 (76.7 %)	33 (70.2 %)	
Oral care (patient)			
+	4 (9.3 %)	2 (4.3 %)	0.338
−	39 (90.7 %)	45 (95.7 %)	
Oral care (institution)			
+	11 (25.6 %)	10 (21.3 %)	0.630
−	32 (74.4 %)	37 (78.7 %)	1

**Table 2:** Treatments for the TJ-14 and placebo groups

	Hange (*n* = 43)	Placebo (*n* = 47)	*p* value
Chemotherapy at the time of registration			
FOLFOX	17 (39.5 %)	19 (40.4 %)	0.752
FOLFIRI	11 (25.6 %)	16 (34.0 %)	
XELOX	4 (9.3 %)	3 (6.4 %)	
Others	11 (25.6 %)	9 (19.1 %)	
Postponed protocol treatment			
+	3 (7.0 %)	4 (8.5 %)	0.786
−	40 (93.0 %)	43 (91.5 %)	
Postponed secondary treatment			
+	8 (19.0 %)	10 (21.7 %)	0.755
−	34 (81.0 %)	36 (78.3 %)	

### Incidence and severity of oral mucositis

During the test treatment cycle, the incidence of WHO grade ≧2 mucositis was lower for patients treated with TJ-14 compared to those treated with placebo (48.8 %(21/43) vs 57.4 %(27/47); relative risk, 0.85, 95 % CI 0.57–1.26), which corresponds to a 15 % risk reduction with TJ-14. However, the difference was not statistical (*p* = 0.41) in terms of the effect of TJ-14 in reducing severity of mucositis.

### Duration of grade ≧2 oral mucositis

**Fig. 1:**
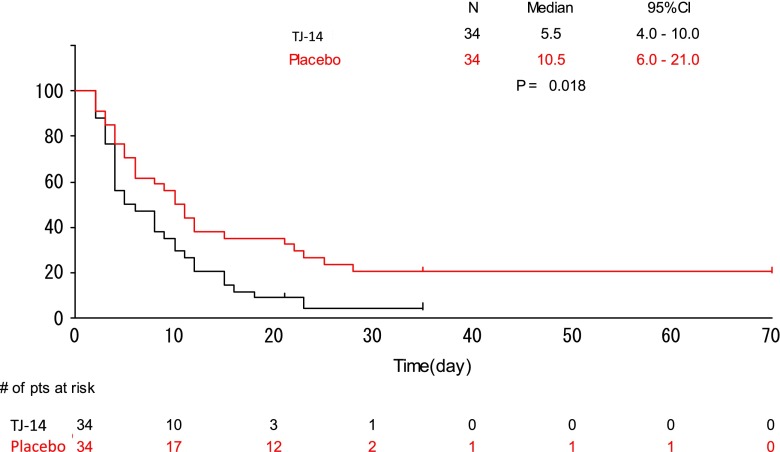
Duration of grade ≧2 mucositis between the treatment groups

The median duration of grade ≧2 oral mucositis was 5.5 days in the TJ-14 group and 10.5 days in the placebo group (*p* = 0.018) (Fig. 1). Treatment with TJ-14 was associated with a statistically significant reduction in the duration of severe grade ≧2 oral stomatitis compared to patients receiving placebo.

### Safety

Hematological, blood biochemistry, and nonhematological toxicities were analyzed. The most commonly reported treatment adverse events including nausea, anorexia, leukopenia, anemia, slight liver dysfunction, and diarrhea, all of which typically occur in cancer patients receiving cytotoxic chemotherapy (Tables 3, 4). The majorities of these events were mild to moderate in severity and considered unrelated to the study drug.

**Table 3:** Hematological and biochemical toxicities observed during treatment

	Grade ≧1			Grade ≧2		
	TJ-14 (*n* = 43)	Placebo (*n* = 47)	*p* value	TJ-14 (*n* = 43)	Placebo (*n* = 47)	*p* value
Leukopenia	2 (4.7 %)	1 (2.1 %)	0.505	0 (0.0 %)	0 (0.0) %)	1.000
Neutropenia	1 (2.3 %)	1 (2.1 %)	0.949	0 (0.0 %)	1 (2.1 %)	0.336
Hb	23 (53.5 %)	20 (42.6 %)	0.300	0 (0.0 %)	2 (4.3 %)	0.171
PLT	9 (20.9 %)	10 (21.3 %)	0.968	0 (0.0 %)	0 (0.0) %)	1.000
T-Bill	0 (0.0 %)	2 (4.3 %)	0.171	0 (0.0 %)	0 (0.0) %)	1.000
AST	7 (16.3 %)	9 (19.1 %)	0.722	0 (0.0 %)	0 (0.0) %)	1.000
Hemorrhage	1 (2.3 %)	1 (2.1 %)	0.949	0 (0.0 %)	0 (0.0) %)	1.000

**Table 4:** Nonhematological toxicities observed during the projected treatment

	Grade ≧1			Grade ≧2		
	TJ-14 (*n* = 43)	Placebo (*n* = 47)	*p* value	TJ-14 (*n* = 43)	Placebo (*n* = 47)	*p* value
Anorexia	20 (46.5 %)	18 (38.3 %)	0.431	2 (4.7 %)	4 (8.5 %)	0.463
Nausea	12 (27.9 %)	18 (38.3 %)	0.296	1 (2.3 %)	3 (6.4 %)	0.351
Vomiting	2 (4.7 %)	2 (4.3 %)	0.927	0 (0.0 %)	0 (0.0 %)	1.000
Diarrhea	9 (20.9 %)	9 (19.1 %)	0.833	1 (2.3 %)	1 (2.1 %)	0.949
Constipation	4 (9.3 %)	8 (17.0 %)	0.282	0 (0.0 %)	0 (0.0 %)	1.000
Peripheral neuropathy	8 (18.6 %)	4 (8.5 %)	0.159	1 (2.3 %)	1 (2.1 %)	0.949
Numbness	2 (4.7 %)	1 (2.1 %)	0.505	0 (0.0 %)	0 (0.0 %)	1.000
Lassitude	3 (7.0 %)	2 (4.3 %)	0.573	0 (0.0 %)	0 (0.0 %)	1.000
Hand-foot syndrome	5 (11.6 %)	4 (8.5 %)	0.622	0 (0.0 %)	1 (2.1 %)	0.336
Skin reaction	2 (4.7 %)	1 (2.1 %)	0.505	1 (2.3 %)	0 (0.0 %)	0.293
Abdominal pain	1 (2.3 %)	0 (0.0 %)	0.293	0 (0.0 %)	0 (0.0 %)	1.000
Abnormal taste	3 (7.0 %)	0 (0.0 %)	0.066	0 (0.0 %)	0 (0.0 %)	1.000
Itchiness	0 (0.0 %)	1 (2.1 %)	0.336	0 (0.0 %)	0 (0.0 %)	1.000
Change in PS	0 (0.0 %)	0 (0.0 %)	1.000	0 (0.0 %)	0 (0.0 %)	1.000

## Discussion

Kampo medicine or Japanese/Chinese traditional herbal medicine has been used for the treatment of various diseases for over 2000 years, mainly in many Asian countries. Although the beneficial effect of those Kampo medicines has long been taken for granted, and was widely utilized among Asian populations, two substantial factors have precluded their approval in the modern western world. One reason is that Kampo medicines are the mixture of several ingredients, and it was believed that combination of those ingredients could exert a synergistic effect. Since analysis of the efficacy of each ingredient is imperative in modern science, attempts to elucidate their mechanisms have not been fully successful to date. Another reason is that the reports on the clinical effectiveness of those Kampo medicines used to be mostly anecdotal, and reliable well-conducted clinical trials to elucidate the evidence of the efficacy have not been satisfactory. In recent years, Asian investigators have attempted to clarify the mechanism and clinical efficacy of the Kampo medicine. The anti-inflammatory effect of Inchinkoto (ICKT) in Wistar rats was proved to improve mortality by ischemic reperfusion of the liver, caused by extensive hepatectomy [20]; Daikenchuto (DKT) showed its effects in revitalizing bowel movement after laparotomy operations in 30 patients [21]. Also, the efficacy of Goshajinkigan (GJG) to alleviate neuropathy after oxaliplatin treatment for colon cancers was investigated in a placebo-controlled randomized phase II trial, and promising results have recently been reported [22].

TJ-14, also one of the Kampo formulas in Japanese traditional herbal medicine, is the mixture of seven herbs including pinellia tuber, scutellaria root, glycyrrhiza, jujube, ginseng, processed ginger, and Coptis rhizome, which are registered in the Japanese Pharmacopoeia XV [23]. In clinical practice, TJ-14 has been used for acute or chronic diarrhea, acute gastroenteritis, and for chronic hypoperfusion of the gastrointestinal system [24, 25]. It is reported that TJ-14 is effective against diarrhea which is the side effect of chemotherapeutic agents for gastrointestinal cancers [26]. Since TJ-14 contains baicalin, an inhibitor of β-glucuronidase, a randomized trial for advanced small cell lung cancer demonstrated the effect of TJ-14 in significantly controlling grade ≧3 diarrhea caused by cisplatin/irinotecan treatment with a *p* value of 0.018 [27, 28]. Moreover, recently, TJ-14 has attracted attention not only for its effect on intestinal mucosa but also concerning its efficacy on oral mucositis. Promising results were published both in vivo [18] and in clinical studies with a topical application of TJ-14 at the ulcerated site of the oral cavity [19].

The present study was conducted to clarify the effect of TJ-14 in patients who developed grade ≧1 mucositis after the first course of chemotherapy for colorectal cancer. With the advent of prospectively randomized placebo–controlled clinical trial, results showing a significant effect of TJ-14 over placebo in terms of the duration of oral mucositis could be a robust evidence for the efficacy of TJ-14. Historically, management of oral mucositis in cancer patients has been limited to supportive care, including pain control, nutritional support, hydration, and wound care. Simple nonspecific interventions such as cryotherapy and compound mouthwashes that include a topical and local anesthetic agent were also shown to have some benefit. Although these interventions may be beneficial, they are not directed to the fundamental mechanism changes associated with the pathophysiology of oral mucositis. The current paradigm for mucosal injury in cancer patients, who underwent 5-FU-based chemotherapy, is based on a complex cascade of mucosal tissue changes that appear to be initiated within hours of exposure to cytotoxic agents [29, 30].

Previously, there was some evidence to recommend the use of benzydamine mouthwash　or misoprostol mouthwash for the prevention of oral mucositis in head and neck cancer patients receiving radiation therapy [31, 32]. Benzydamine hydrochloride is a nonsteroidal anti-inflammatory drug (NSAID), which has been shown to inhibit inflammatory cytokine production, such as TNF-α and IL-1β. Misoprostol is a synthetic analog of prostaglandin E1 (PGE1), which has anti-inflammatory properties [33, 34]. Misoprostol also protects the mucosa and has been approved for reducing the risk of gastric ulcers induced by NSAID use [32, 35]. Although positive results were reported for some other anti-inflammatory agents in the patients who received radiation therapy, no guidelines were able to be developed for any other agents due to insufficient and/or conflicting evidence for COM.

In conclusion, the present study results did not meet the primary endpoint. However, TJ-14 demonstrated a significant effect in the treatment of grade ≧2 mucositis in patients with colorectal cancer compared to the placebo. Another extensive randomized placebo-controlled phase III study to reconfirm the effect of TJ-14 is warranted.

## Appendix

In addition to the authors listed in the title page, the following investigators and institutions contributed equally to this study. Michiya Kobayashi: Kochi University, Yoshiaki Shindo: Nakadori General Hospital, Yasuhiro Miyake: Mino Municipal Hospital, Hiroaki Takemoto: Sakai Municipal Hospital, Kenji Kobayashi: Matusnami General Hospital, Keiichiro Ishibashi: General Medical Center Saitama Medical College, Hitoshi Inagaki: Gifu Chuo Hospital, Shinya Morimoto: Tokushima University, Yukihiko Tokunaga: Kyoto Teishin Hospital, Junichi Hasegawa: Osaka Rosai Hospital.
